# Exploration of the Role of Serine Proteinase Inhibitor A3 in Alcohol Dependence Using Gene Expression Omnibus Database

**DOI:** 10.3389/fpsyt.2021.779143

**Published:** 2022-01-12

**Authors:** Bo Zhang, Gang Wang, Cheng Bing Huang, Jian Nan Zhu, Yong Xue, Jian Hu

**Affiliations:** ^1^Department of Psychiatry, The First Affiliated Hospital of Harbin Medical University, Harbin, China; ^2^Department of Substance Dependence, Wuhan Mental Health Center, Wuhan, China; ^3^The Third People's Hospital of Huai'an, Huai'an, China

**Keywords:** alcohol dependence, bioinformatics analysis, differently expressed genes, SERPINA3, relapse biomarkers

## Abstract

**Background:** Alcohol dependence is an overall health-related challenge; however, the specific mechanisms underlying alcohol dependence remain unclear. Serine proteinase inhibitor A3 (*SERPINA3*) plays crucial roles in multiple human diseases; however, its role in alcohol dependence clinical practice has not been confirmed.

**Methods:** We screened Gene Expression Omnibus (GEO) expression profiles, and identified differentially expressed genes (DEGs). Protein-protein interaction (PPI) networks were generated using STRING and Cytoscape, and the key clustering module was identified using the MCODE plugin. *SERPINA3*-based target microRNA prediction was performed using online databases. Functional enrichment analysis was performed. Fifty-eight patients with alcohol dependence and 20 healthy controls were recruited. Clinical variables were collected and follow-up was conducted for 8 months for relapse.

**Results:** *SERPINA3* was identified as a DEG. *ELANE* and miR-137 were identified after PPI analysis. The enriched functions and pathways included acute inflammatory response, response to stress, immune response, and terpenoid backbone biosynthesis. *SERPINA3* concentrations were significantly elevated in the alcohol dependence group than in healthy controls (*P* < 0.001). According to the median value of *SERPINA3* expression level in alcohol dependence group, patients were divided into high *SERPINA3* (≥2677.33 pg/ml, *n* = 29) and low *SERPINA3* groups (<2677.33 pg/ml, *n* = 29). Binary logistic analysis indicated that IL-6 was statistically significant (*P* = 0.015) Kaplan-Meier survival analysis did not indicate any difference in event-free survival between patients with low and high *SERPINA3* levels (*P* = 0.489) after 8 months of follow-up. Receiver characteristic curve analysis revealed that *SERPINA3* had an area under the curve of 0.921 (*P* < 0.0001), with a sensitivity and specificity of 93.1 and 80.0%, respectively. Cox regression analysis revealed that aspartate transaminase level was a negative predictor of relapse (β = 0.003; hazard ratio = 1.003; *P* = 0.03).

**Conclusions:** *SERPINA3* level was remarkably elevated in patients with alcohol dependence than healthy controls, indicating that *SERPINA3* is correlated with alcohol dependence. However, *SERPINA3* may not be a potential predictive marker of relapse with patients in alcohol dependence.

## Introduction

Alcohol dependence is associated with physiological and psychological effects, with alcohol abuse being the most common cause of many social issues, including domestic violence and other crimes. Several studies have shown that abnormal gene expression and polymorphism are strongly associated with alcohol dependence. Several intronic γ-aminobutyric acid β1 (GABA β1) subunit (GABR β1)single nucleotide polymorphisms that may directly influence alcohol dependence risk have been recently identified (1). DNA methylation of growth arrest specific five gene is implicated in alcohol use (2). In addition, carriers of the rs1789891 A allele reportedly consume more alcohol and have a higher risk of relapse than individuals that are homozygous for the C allele (3). However, there is currently no effective diagnostic and relapse test to evaluate alcohol dependence. The systemic molecular mechanisms of patients with alcohol dependence must be urgently explored to provide new, effective diagnostics and treatments.

Bioinformatics has become an essential tool in life science research, and plays an important role in studying the identification and functional annotation of human genes and proteins. In particular, in recent years, bioinformatics has played a pivotal role in the study of human diseases and related mechanisms, including gene expression regulation analysis, drug screening and targeting, and the formulation and validation of biological hypotheses, using the vast data resources available in public databases. However, the reliability of the results is challenging because the false-positive rate in independent microarray analysis remains high. To this end, we aimed to explore the underlying molecular mechanisms among patients with alcohol dependence using combined bioinformatics. In the present study, three alcohol-related gene expression omnibus (GEO) datasets were used to identify differentially expressed genes (DEGs) that may be associated with alcohol dependence. Functional enrichment analysis of these DEGs was then used to discover the underlying biological mechanisms of alcohol dependence. These results were verified using an independent cohort of patients with alcohol dependence and healthy controls. Our study findings could contribute toward developing a clinical diagnostic test for alcohol dependence, and uncovering new therapeutic targets to combat this disease.

## Methods

### Microarray Data

We obtained data from three gene expression datasets: GSE29555 (4), GSE44456 (5), and GSE62699 (6) from the GEO database. The GSE29555 dataset contained 68 samples from alcohol-dependent patients and 60 from non-alcohol-dependent patients. GSE44456 included 19 samples from alcohol-dependent patients and 19 from non-alcohol-dependent patients. GSE62699 contained 18 samples from alcohol-dependent patients and 18 from non-alcohol-dependent patients.

### DEG Validation

GEO2R tool was utilized to identify the DEGs between the samples of patients with alcohol dependence and non-alcohol dependence. Probe sets lacking matched gene symbols, and the datasets that GEO2R could not analyze were removed from the analysis among 20 datasets. DEGs with |log FC (fold change)| ≥ 1 and Statistical significance was considered *p* < 0.05.

### Protein-Protein Interaction Network Analysis

A protein-protein interaction (PPI) network of the DEGs was constructed using the STRING database (http://string-db.org; version 10.0), with a minimum interaction score cutoff of 0.4 (7). Cytoscape (version 3.7.2) was used for visualization of the interaction networks (8). The MCODE plug-in was used to construct key clustering modules (MCODE score > 5, degree cutoff = 2, node score cutoff = 0.2, max depth = 100, and k-score = 2).

### Functional Enrichment Analysis

Functional and pathway enrichment analyses of the DEGs were performed using DAVID (https://david.ncifcrf.gov/) (9). Gene ontology (GO) analysis was performed to associate functional keywords with the DEGs in the following categories: biological process (BP), molecular function (MF), and cellular component (CC) (10).

### Clinical Samples

To verify the hypothesis generated using the bioinformatics data, a cohort of 58 patients and 20 healthy controls was recruited from the Third People's Hospital of Huai'an. All participants provided written informed consent. The study was authorized and approved by the Harbin Medical University Ethics Committee and the Third People's Hospital Ethics Committee of Huai'an. All patients were diagnosed by Diagnostic and Statistical Manual of Mental Disorders-Fifth Edition (DSM-V). Sociodemographic and clinical variables were collected for all participants, and Montreal cognitive assessment (MoCA) was used to test the cognitive function in patients with alcohol dependence.

### Sample Collection and Processing

All blood samples were obtained before 07:00 AM, when the patients were first admitted to the hospital and before any medication was given. The samples were immediately centrifuged at 3,000 g for 15 min at ambient temperature. The plasma supernatant was removed and stored at −80°C. The concentration of *SERPINA3* in each plasma sample was assayed using an ELISA kit (SERPINA3 Human ELISA Kit, YanZun, Shanghai, China) as per the manufacturer's protocol. We also measured the levels of IL-6, which interacts with *SERPINA3*, using an ELISA kit (IL-6 Human ELISA Kit, YanZun, Shanghai, China).

### Statistics

The Kolmogorov-Smirnov test was used to test normal distribution. Continuous variables were expressed as the mean ± standard deviation. Student's *t*-test was used to analyze the continuous variables. Categorical variables were analyzed using the chi-squared test or Fisher's exact test. Binary logistic analysis was performed to identify variables that were independently influenced by *SERPINA3* levels. Multivariate linear regression analysis were used to determine the variables most closely related to *SERPINA3* levels. Clinical events were systematically tracked for the entire cohort using post-discharge telephone interviews to document relapse. Receiver operating characteristic (ROC) curves and the area under the curve (AUC) were analyzed to test the diagnostic value of *SERPINA3* for alcohol dependence. Kaplan-Meier survival curves were generated to compare the time of relapse to *SERPINA3* levels between the high and low *SERPINA3* groups. Cox regression analysis was used to determine the risk factors affecting first-time relapse. Statistical analyses were performed in SPSS-23 (IBM) and graphs were generated in Origin (Origin 2018 64Bit). Differences between the groups were considered statistically significant at *P* < 0.05.

## Results

### Identification of DEGs Associated With Alcohol Dependence

**Figure 1:**
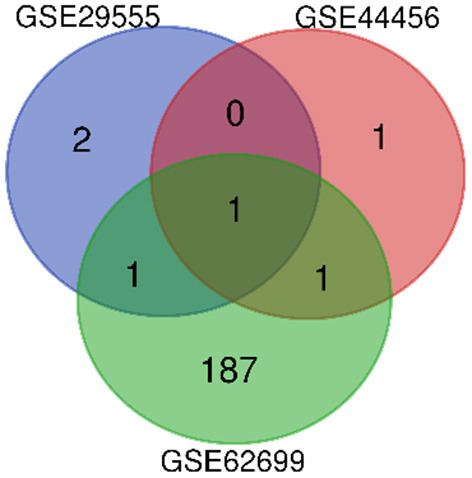
Venn diagram showing DEGs between three microarray databases for genes associated with alcohol dependence. The three datasets have one overlapping gene, *SERPINA3*.

DEGs between patients with alcohol dependence and those without alcohol dependence were identified (4 in GSE29555, 3 in GSE44456, and 190 in GSE62699). One overlapping DEG was identified in all three datasets, and this DEG was upregulated in all three datasets (Figure 1).

### PPI Network Analysis

**Figure 2:**
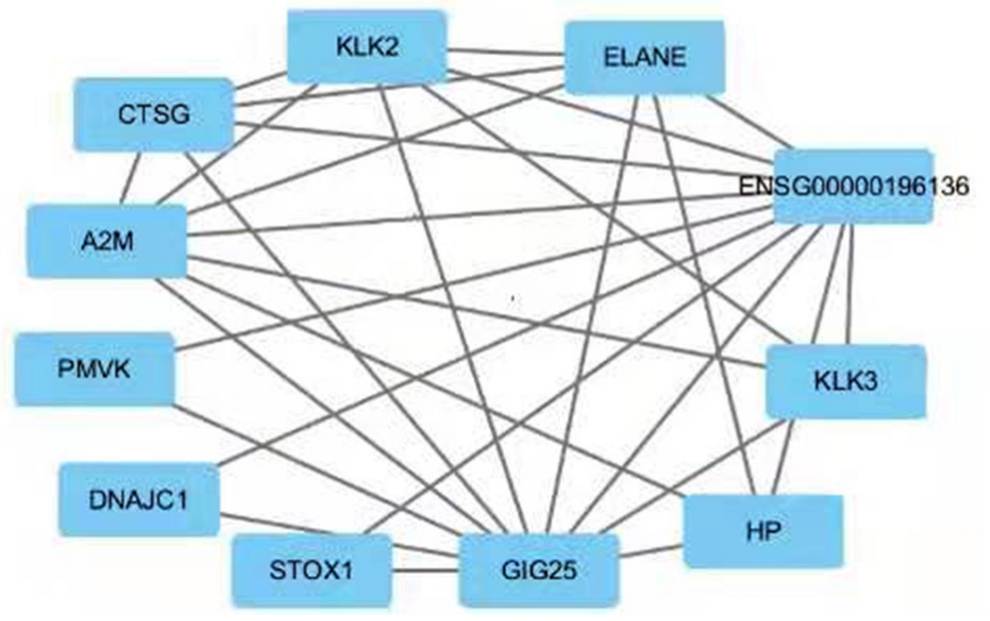
PPI network for SERPINA3. The interacting genes were identified using the STRING database and visualized using Cytoscape.

**Figure 3:**
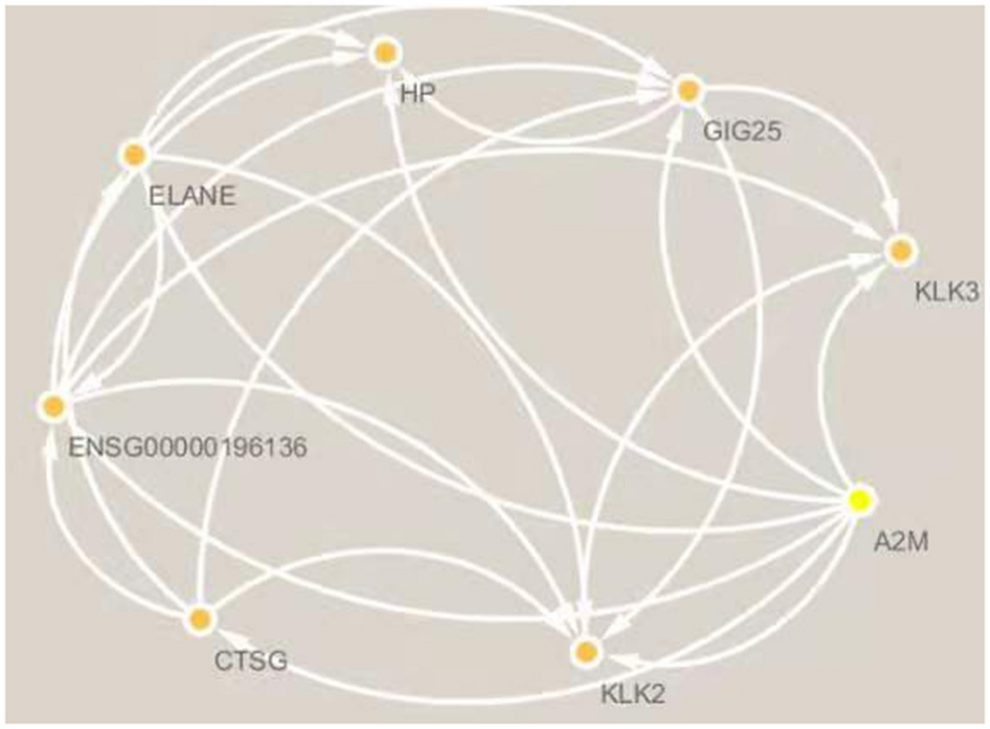
Clustering module 1 scored 6.571 and had 8 nodes and 23 edges. The hub gene is *ELANE*.

Since only a single common gene was identified, we generated a network of interacting proteins to augment the dataset (Figure 2). MCODE plug-in was used to analyze this network, and one clustering module was filtered out according to the chosen screening conditions. Clustering module 1 scored 6.857 and had 8 nodes and 24 edges (Figure 3). One gene, *ELANE*, was identified as the hub gene with degree ≥ 10.

### SERPINA3 Target Gene Prediction

**Figure 4:**
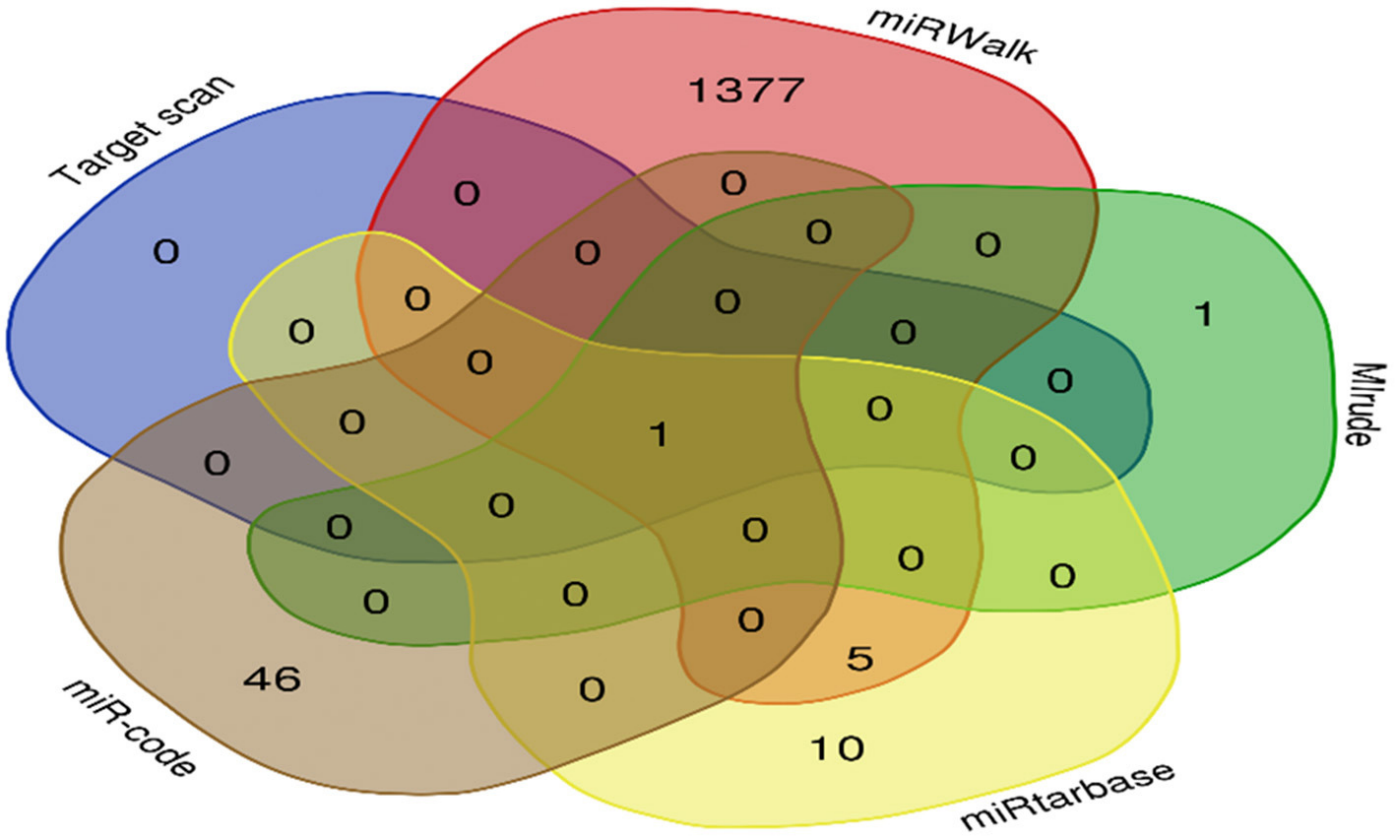
SERPINA3 target gene prediction. The five datasets had one overlapping microRNA, miR-137.

MicroRNAs of the target gene were predicted from TargetScan (http://www.targetscan.org/), miRTarBase (https://maayanlab.cloud/Harmonizome/resource/MiRTarBase), miRWalk (mirwalk.umm.uni-heidelberg.de/), miRcode (http://www.mircode.org), and miRDB (http://mirdb.org/miRDB/) databases. To narrow the range of the predicted miRNAs and reduce the false positive rate, miRNA-137, the intersection of miRNAs obtained from the five databases, was taken as the prediction result of the target genes (Figure 4).

### Functional Enrichment Analysis of the DEGs

The DEG were significantly enriched in the following biological processes: acute inflammatory response, defense response, neutrophil-mediated immunity, acute-phase response, negative regulation of protein metabolic process, negative regulation of chemokine biosynthetic response to external stimulus, positive regulation of immune response, immune response, and humoral immune response. The enriched MFs were: serine-type endopeptidase activity, and enzyme regulator activity. The enriched CCs were: extracellular space, secretory granules, and extracellular exosomes. Kyoto encyclopedia of genes and genomes (KEGG) pathway analysis revealed that genes were enriched mainly in terpenoid backbone biosynthesis (*P* = 0.022).

### Experimental Validation of Bioinformatics Results

**Figure 5:**
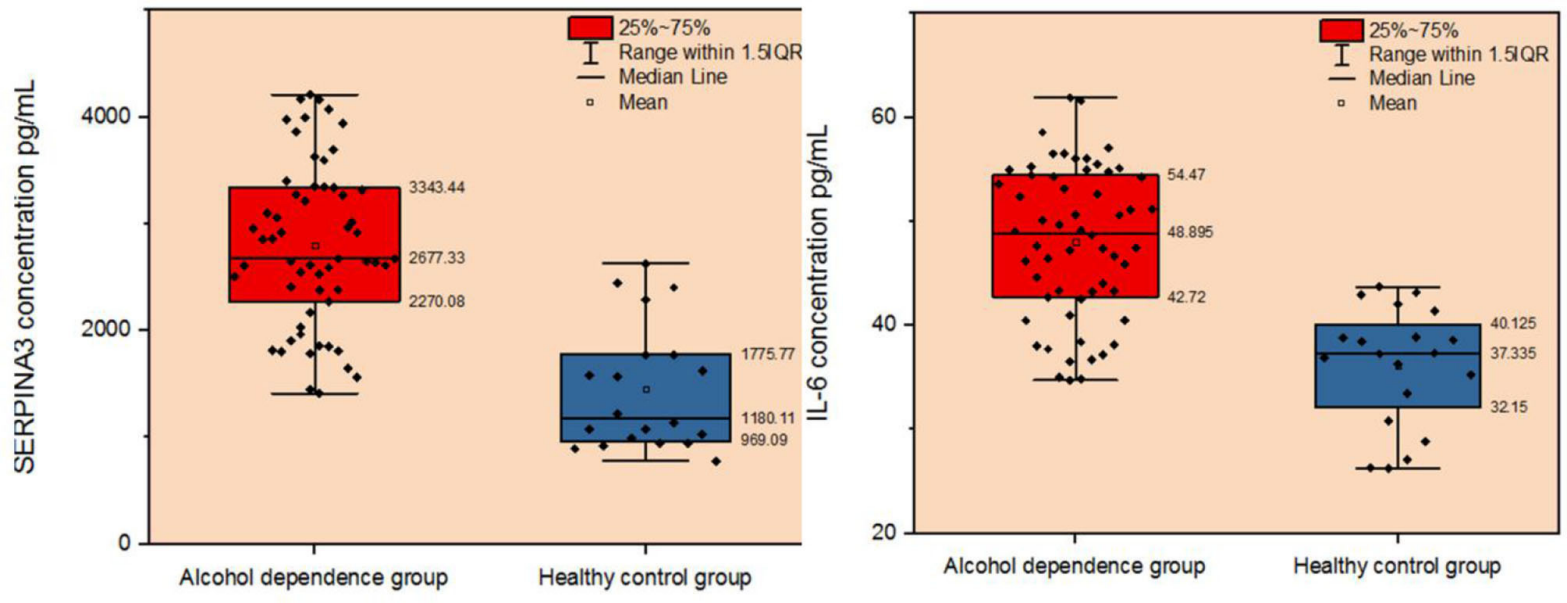
Comparison of plasma SERPINA3 and IL-6 levels between patients with alcohol dependence and the healthy control group.

We recruited 58 individuals with alcohol dependence and 20 healthy controls to validate the hypothesis that *SERPINA3* and IL-6 were dysregulated in individuals with alcohol dependence. ELISA analysis indicated that SERPINA3 and IL-6 concentrations were significantly elevated in patients with alcohol dependence than in healthy controls (*P* < 0.001) (Figure 5). The median *SERPINA3* concentration in patients with alcohol dependence (*n* = 58) was 2677.33 pg/ml. According to the median level of *SERPINA3* in this cohort, patients were divided into high *SERPINA3* (≥2677.33 pg/ml, *n* = 29) and low *SERPINA3* groups (<2677.33 pg/ml, *n* = 29). Table 1 shows the comparison of the baseline data between the two groups. Higher concentrations of *SERPINA3* were associated with higher concentrations of IL-6 (*P* = 0.005). In contrast, higher *SERPINA3* concentration was associated with lower leukocyte and neutrophil counts (*P* = 0.023 and *P* = 0.038, respectively). Binary logistic analysis indicated that IL-6 was statistically significant (*P* = 0.015) (Table 2).

**Table 1:** Correlation between basic clinical information, laboratory tests, and SERPINA3 levels in the plasma of patients recruited for the study.

	**High SERPINA3 level (*n* = 29)**	**Low SERPINA3 level (*n* = 29)**	***P*-value**
**Demographics**			
Age, years	45 ± 11	44 ± 10	0.540
Male, *n* %	29 (100)	28 (96.6)	1.00
Education (year)			1.00
≤ 6	4 (13.8%)	5 (17.2%)	
6–12	21 (72.4%)	21 (72.4%)	
≥12	4 (13.8%)	3 (10.3%)	
Marital status, *n* (%)			0.530
Unmarried	5 (17.2%)	3 (10.3%)	
Married	20 (69.0%)	19 (65.5%)	
Divorced	4 (13.8%)	7 (24.1%)	
Occupation, *n* (%)			0.654
Mental labor	4 (13.8%)	3 (10.3%)	
Physical labor	12 (41.4%)	9 (31.0%)	
Unemployed	13 (44.8%)	17 (58.6%)	
BMI, kg/m^2^	23.29 ± 3.44	22.30 ± 3.03	0.251
Drinking duration (year)	19 ± 10	17 ± 9	0.315
Heart rate, bpm	97.17	12.58	0.101
Systolic blood pressure, mmHg	134 ± 17	138 ± 16	0.351
Diastolic blood pressure, mmHg	89 ± 11	91 ± 10	0.427
Hypertension, *n* (%)	6 (20.7%)	6 (20.7%)	1.00
Diabetes mellitus, *n*, (%)	2 (6.9%)	1 (3.4%)	1.00
Current smoker, *n*, (%)	26 (89.7%)	25 (86.2%)	1.00
**Laboratory tests**			
RBC	7.51 ± 18.65	4.32 ± 0.50	0.362
Hemoglobin, g/L	136.24 ± 25.52	144.48 ± 13.19	0.130
MCV	103.66 ± 9.04	99.91 ± 5.83	0.065
Leukocytes, ×10^9^/L	6.89 ± 2.58	8.60 ± 3.00	0.023
Neutrophil, ×10^9^/L	4.81 ± 2.29	6.26 ± 2.89	0.038
Lymphocyte, ×10^9^/L	1.59 ± 0.68	1.76 ± 0.81	0.390
Platelets, ×10^9^/L	200.54 ± 80.85	199.23 ± 64.73	0.946
AST, U/L	117.48 ± 144.88	62.69 ± 55.26	0.065
ALT, U/l	50.17 ± 45.81	38.52 ± 27.92	0.247
GGT, U/L	299.38 ± 399.95	216.90 ± 326.70	0.393
TBIL, μmol/L	23.48 ± 22.77	21.76 ± 16.35	0.743
DBIL, μmol/L	9.60 ± 11.83	7.05 ± 5.41	0.295
IBIL, μmol/L	11.63 ± 7.60	11.18 ± 7.74	0.823
Total cholesterol, mmol/L	6.08 ± 4.47	5.05 ± 2.29	0.274
HDL, mmol/L	1.52 ± 0.64	1.65 ± 0.50	0.377
LDL, mmol/L	2.69 ± 1.08	2.38 ± 0.92	0.247
Triglycerides, mmol/L	3.19 ± 5.98	1.94 ± 2.86	0.315
Serum creatinine, μmol/L	57.63 ± 19.31	62.10 ± 18.00	0.365
UA, μmol/L	392.49 ± 106.93	404.20 ± 163.69	0.748
BUN, mmol/L	4.21 ± 2.18	4.29 ± 2.22	0.896
Fasting glucose, mmol/L	5.88 ± 2.05	5.97 ± 2.71	0.883
CK, U/L	723.67 ± 1908.74	354.18 ± 463.93	0.319
CK-MB, U/L	19.74 ± 20.20	14.94 ± 7.00	0.235
C-reactive protein, mg/L	15.28 ± 38.12	8.67 ± 13.76	0.386
IL-6, pg/ml	50.70 ± 7.11	45.46 ± 6.58	0.005
MoCA, *n* (%)			0.770
≥26	22 (75.9%)	7 (24.1%)	
<26	20 (69.05%)	9 (31.0%)	

*BMI, body mass index; RBC, red-blood-cell; MCV, mean corpuscular volume; AST, aspartate aminotransferase; ALT, alanine transaminase; GGT, gamma-glutamyl transpeptidase; TBIL, total bilirubin; DBIL, direct bilirubin; IBIL, indirect bilirubin; HDL, high-density lipoprotein; LDL, low-density lipoprotein; UA, uric acid; BUN, blood urea nitrogen; CK, creatine kinase; CK-MB, creatine kinase MB; IL-6, interleukin-6; MoCA, Montreal cognitive assessment*.

**Table 2:** Binary logistic regression models for SERPINA3.

	**B**	**S.E**.	**Sig**.	**OR**	**95% CI**	
					**Lower**	**Upper**
IL-6	−0.105	0.043	0.015	0.900	0.827	0.980
Neutrophil	−0.218	0.410	0.596	0.804	0.360	1.797
WBC	0.405	0.388	0.297	1.499	0.700	3.208
Constant	3.113	2.353	0.186	22.490		

*WBC, white blood cell; CI, confidence ration; OR, odds ratio; SE, standard error*.

### Correlation and Linear Regression Analysis of *SERPINA3* Levels

Plasma *SERPINA3* levels correlated positively with IL-6 levels (r = 0.357, *P* = 0.006), whereas it correlated negatively with white blood cell (r = −0.442, *P* = 0.001) and neutrophil counts (r = −0.441, *P* = 0.001). Multivariate linear regression analysis revealed that IL-6 (standardized β = 0.299, *p* = 0.013) was an independent determinant of *SERPINA3* levels (Table 3).

**Table 3:** Linear regression models for SERPINA3.

**Variable**	**Univariate analysis**		**Multivariate analysis**	
	**Standardized** **β** ***P*****-value**		**Standardized** **β** ***P*****-value**	
IL-6	0.357	0.006	0.299	0.013
WBCs	−0.442	0.001	−0.225	0.605
Neutrophils	−0.441	0.001	−0.181	0.677

*WBC, white blood cell*.

### Plasma *SERPINA3* as a Predictor of Relapse

**Figure 6:**
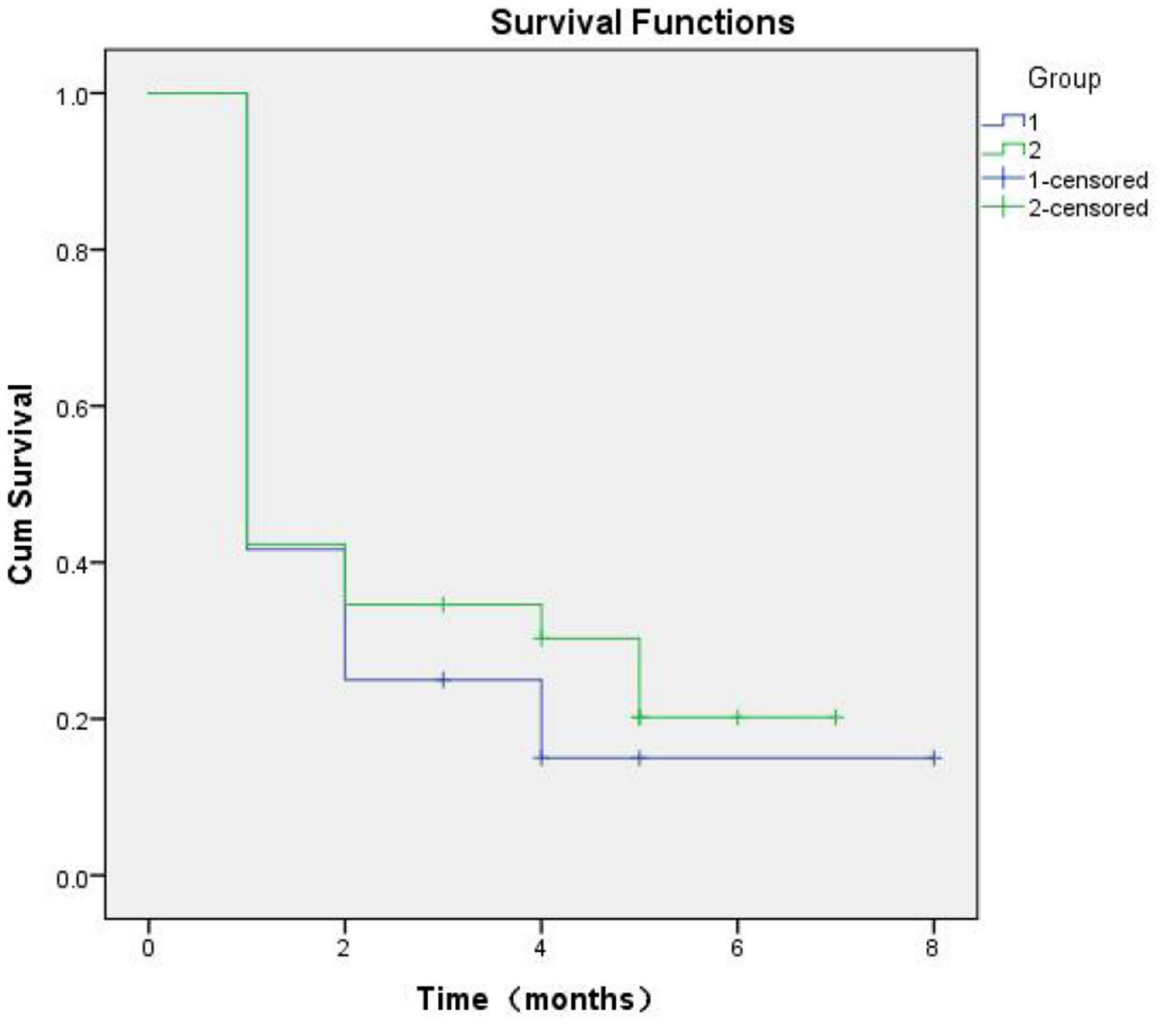
Kaplan-Meier curves based on SERPINA3 levels in patients with alcohol dependence during follow-up of up to 8 months (*P* = 0.489).

**Figure 7:**
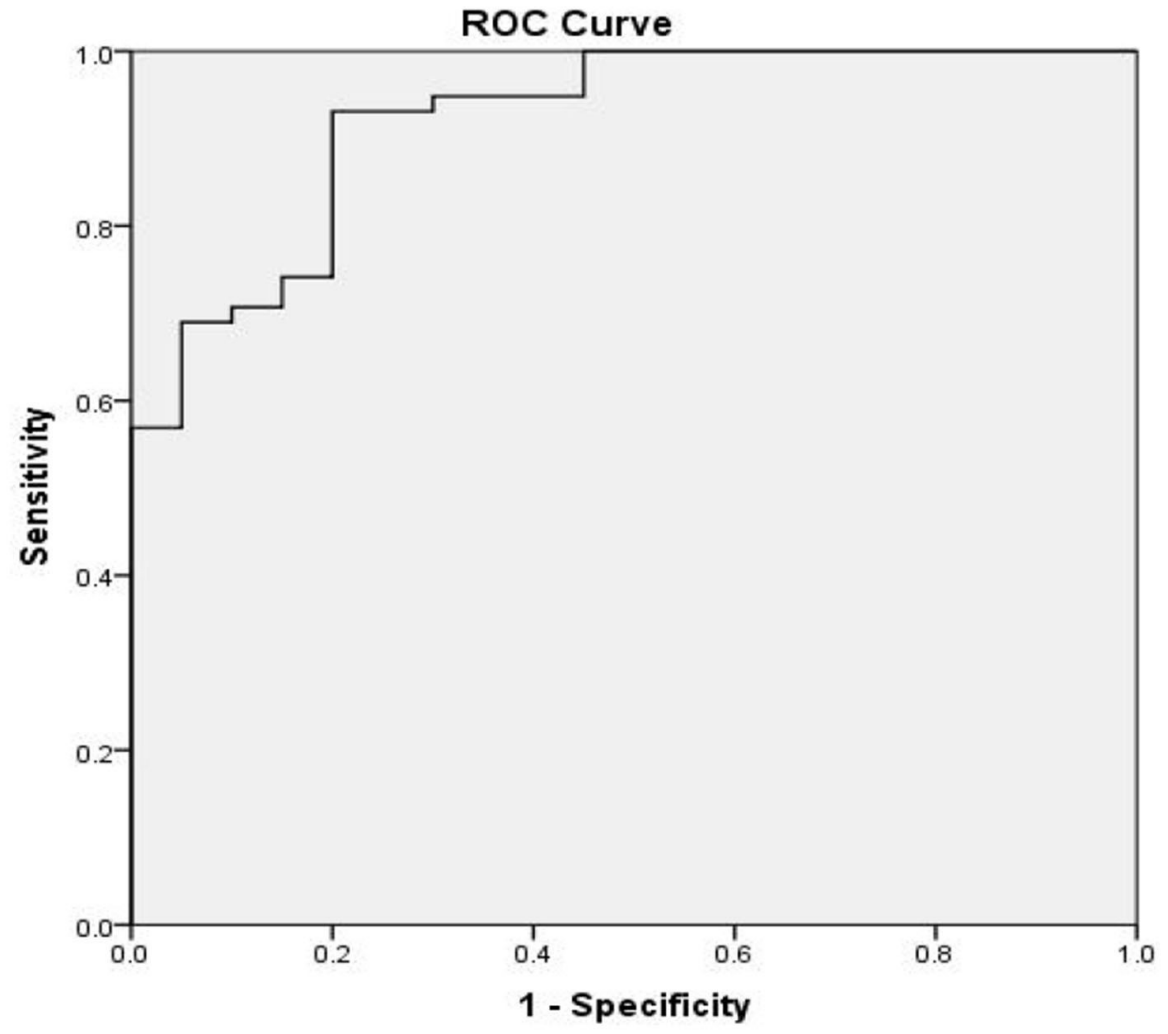
Receiver operator characteristic (ROC) curve based on SERPINA3 levels in patients with alcohol dependence. The area under the curve (AUC) for SERPINA3 was 0.921 (*P* < 0.0001), sensitivity was 93.1%, and specificity was 80.0%.

At 8 months of follow-up, Kaplan-Meier survival analysis did not reveal any difference in the event-free survival between patients with low vs. high *SERPINA3* levels (*P* = 0.489) (Figure 6). ROC analysis was used to test the diagnostic value of *SERPINA3*, wherein it revealed that *SERPINA3* had an AUC of 0.921(*P* < 0.0001), sensitivity of 93.1%, and specificity of 80.0% (Figure 7). Cox regression analysis showed that aspartate transaminase (AST) level was a negative predictor of relapse (β = 0.003, hazard ratio = 1.003, *P* = 0.03), whereas *SERPINA3* level was not a predictor of relapse (Table 4).

**Table 4:** Cox regression analyses for predictors of relapse.

**Variable**	**Univariate analysis**		**Multivariate analysis**	
	**HR (95% CI)** ***P*****-value**		**HR (95% CI)** ***P*****-value**	
AST	1.004	0.017	1.003	0.030
Lymphocyte	1.594	0.042	1.485.	0.070

*AST, aspartate transaminase; CI, confidence interval; HR, hazard ratio*.

## Discussion

**Figure 8:**
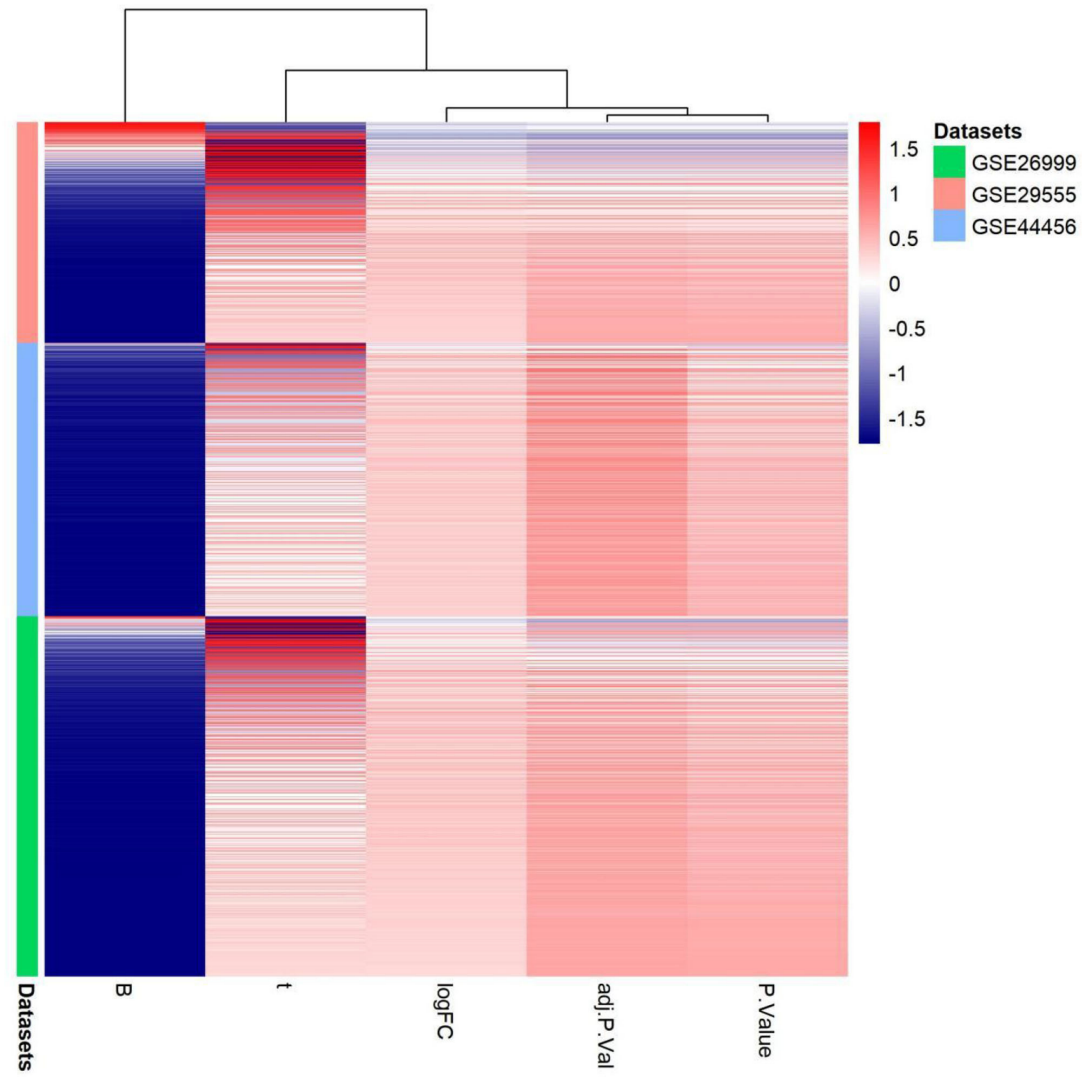
Gene expression patterns in the three gene expression datasets: GSE29555, GSE44456, and GSE62699. Upregulation of genes is marked in red; downregulation of genes is marked in blue.

Alcohol dependence is a major public health issue associated with increasing incidence and mortality. The main etiology of alcohol dependence includes genetic and environmental factors, with genetic factors accounting for 40–60% (11). Most studies have been conducted on immune inflammation (12, 13), oxidative stress (14), metabolism (15), and apoptosis of brain cells (16) in patients with alcohol dependence. Despite ongoing studies on the pathology, causes, and risk factors of this disease, an appropriate treatment regimen has not yet been determined. Thus, the discovery and validation of the molecular mechanisms underpinning alcohol dependence are urgently needed. Microarray technology has increased our ability to explore genetic alterations in patients with alcohol dependence with several previous studies utilizing microarray data (17). In the present study, we identified a single upregulated DEG, *SERPINA3*, and one hub gene among the three microarray datasets associated with alcohol dependence. We also analyzed the gene expression patterns of three gene expression datasets: GSE29555, GSE44456, and GSE62699 in the Supplementary Material; (Figure 8) PPI analysis determined that genes associated with terpenoid backbone biosynthesis were associated with *SERPINA3*. Dysregulation of the terpenoid backbone biosynthesis plays a crucial role in mediating the effect of Panax notoginseng saponins (PNS) progression (18). Ginsenoside-Rg1, the main component of PNS, significantly suppresses inflammation by alleviating microglia and astrocyte activation (19). Microglia are critical modulators of alcohol neurotoxicity, and microglia may also be closely related to alcohol use disorders (20, 21). *SERPINA3* is produced by brain astrocytes, which may be involved in synaptic remodeling and neuronal survival (22, 23). Therefore, it is possible that ginsenoside-Rg1 could be used to treat alcohol dependence by inhibiting glial cell activation. This promising area of research requires further investigation to evaluate the links between alcohol dependence and ginsenoside-Rg1.

This is the first study to combine bioinformatics with clinical and genetic research on alcohol dependence. We are the first to demonstrate that plasma *SERPINA3* is correlated with alcohol dependence. We additionally identified one hub gene, *ELANE*, and also conducted microRNA target prediction of *SERPINA3*.

*SERPINA3* is a serine protease inhibitor superfamily member, which is involved in apoptotic cell death, oxidative stress, and inflammatory response (24). *SERPINA3* is an acute phase protein that functions primarily through the regulation of neutrophil cathepsin G, leukocyte elastase, and mast cell chymotrypsin during inflammation, and it might be involved in synaptic remodeling and neuronal survival. The gene expression of *SERPINA3* is stimulated by cytokines (25, 26). Leukocyte elastase G has a strong association with *SERPINA3* (27). However, excessive stimulation of *SERPINA3* can lead to tissue damage (28). During alcohol dependence, activated neutrophils could be partially responsible for the elevated levels of *SERPINA3* observed in our study. A previous study demonstrated that *SERPINA3 transcripts* were remarkably expressed in a high inflammation cluster than in a low inflammation cluster (29). Similarly, in the present study, higher *SERPINA3* levels were positively correlated with increased IL-6 level (*P* = 0.005), lower leukocyte count (*P* = 0.023), and lower neutrophil count (*P* = 0.038). This suggests that *SERPINA3* is involved in inflammation during alcohol dependence. The levels of *SERPINA3* were higher in patients with alcohol dependence, which is related to the NF-κB pathway (5, 30). Further research is needed to elucidate the exact mechanisms by which *SERPINA3* levels are associated with alcohol dependence.

Previous studies indicated that peripheral *SERPINA3* levels are not elevated in patients with dementia, other than those with Alzheimer's disease, and increase as dementia progresses (31). Our evidence was consistent with previous studies; we found no correlation between *SERPINA3* levels and MoCA.

*ELANE* is involved in the inflammation response of leukocytes (32). Neutrophil elastase (NE), encoded by the *ELANE* gene, plays a bactericidal and proinflammatory role (33). When NE is released at high concentrations, it can mediate tissue destruction (34). After acute alcoholism, the total number of white blood cells and absolute value of neutrophils continue to increase, possibly leading to systemic or local inflammatory damage in the body (35). Therefore, we speculate that inhibitors of NE can be used to improve survival by attenuating the systemic inflammatory response; however, further validation is needed in this regard.

*SERPINA3*-based target microRNA prediction revealed miR-137. Reportedly, miR-137 plays a key regulator role in adult neurogenesis, presynaptic plasticity, and neuronal maturation (36). A previous study suggests that alcohol intake leads to synapse loss and anxiety-like behavior (20).

Furthermore, miR-137 is also essential for dendritic and synaptic growth. Loss of function of miR-137 leads to altered synaptic plasticity as well as anxiety-like behavior in mice (37). miR-137 overexpression or downregulation can impair brain function (38). Overexpression of miR-137 reduces the extent of brain tissue damage, and improves cell proliferation, and decreases the rate of apoptosis by inhibiting the expression of JAK1 and STAT1 proteins (39). Loss of miR-137 expression leads to abnormal neuronal morphology (40). These findings are in line with our hypothesis regarding the mechanism underlying alcohol dependence. However, the possible role of miR-137 in alcohol dependence has not been elucidated. We consider that miR-137 dysregulation may be associated with alcohol dependence; however, further studies are needed to validate our speculation.

Currently, the theraputic intervention is limited, including cognitive behavioral therapy and rehabilitation treatment approaches, Because of poor treatment compliance, with ~40–60% of patients relapse within 1 year of treatment (41). Therefore, after acute detoxification treatment, determining the causes of relapse and preventing relapse are crucial to treatment success. Previous studies have found that high GGT, AST, ALT, and MCV levels are strongly correlated with alcohol dependence relapse (42, 43). In the present study, AST was the main risk factor for relapse after inpatient withdrawal treatment. Therefore, patients with high-level AST should be paid more attention to prevent them from relapsing. However, we could not identify factors that influenced relapse, possibly because of the small sample size. Here, the baseline *SERPINA3* levels did not differ significantly in patients with alcohol dependence between those who relapsed and those who did not; this suggests that *SERPINA3* is not suitable for predicting relapse in alcohol dependence.

## Conclusion

In conclusion, we identified that *SERPINA3* is correlated with alcohol dependence. It is imperative to conduct further research to elucidate the underlying mechanisms and develop effective therapeutic strategies based on these findings. We speculate that there could be a relationship between ginsenoside-Rg1 and alcohol dependence. Future research should focus on determining the mechanism of action of ginsenoside-Rg1 on alcohol dependence. *ELANE* and miR-137 also need to be validated further as potential therapeutic targets in a larger cohort. However, the present study has several limitations. The cohort under study was small in size and blood samples were collected only at the time of admission; thus, there were no continuous data to track the change in the level of *SERPINA3* during the follow-up.

## Data Availability Statement

The datasets presented in this study can be found in online repositories. The names of the repository/repositories and accession number(s) can be found in the article/supplementary material.

## Ethics Statement

The studies involving human participants were reviewed and approved by Harbin Medical University Ethics Committee and the Third People's Hospital Ethics Committee of Huai'an. The patients/participants provided their written informed consent to participate in this study.

## Author Contributions

BZ and GW designed the study. CH, JZ, and YX collected the data. BZ performed the statistical analysis and wrote the manuscript. JH revised the manuscript. All authors contributed to the article and approved the submitted version.

## Funding

Our study was supported by the National Key R&D Program of China (No. 2018YFC1314400, 2018YFC1314402).

## Conflict of Interest

The authors declare that the research was conducted in the absence of any commercial or financial relationships that could be construed as a potential conflict of interest.

## Publisher's Note

All claims expressed in this article are solely those of the authors and do not necessarily represent those of their affiliated organizations, or those of the publisher, the editors and the reviewers. Any product that may be evaluated in this article, or claim that may be made by its manufacturer, is not guaranteed or endorsed by the publisher.

## Abbreviations

GEO: Gene expression omnibus
DEGs: Differentially expressed genes
AST: Aspartate transaminase
SERPINA3: Serine proteinase inhibitor A3.
