# Corrigendum: Exploration of the Role of Serine Proteinase Inhibitor A3 in Alcohol Dependence Using Gene Expression Omnibus Database

**DOI:** 10.3389/fpsyt.2022.857211

**Published:** 2022-03-15

**Authors:** Bo Zhang, Gang Wang, Cheng Bing Huang, Jian Nan Zhu, Yong Xue, Jian Hu

**Affiliations:** ^1^Department of Psychiatry, The First Affiliated Hospital of Harbin Medical University, Harbin, China; ^2^Department of Substance Dependence, Wuhan Mental Health Center, Wuhan, China; ^3^The Third People's Hospital of Huai'an, Huai'an, China

**Keywords:** alcohol dependence, bioinformatics analysis, differently expressed genes, SERPINA3, relapse biomarkers

In the original article, there was a mistake in Figure 5 as published. We found a problem with the layout of the images. Figure 5 should contain two charts, the Figures 5, 6 in the original article as published.

**Figure 5:**
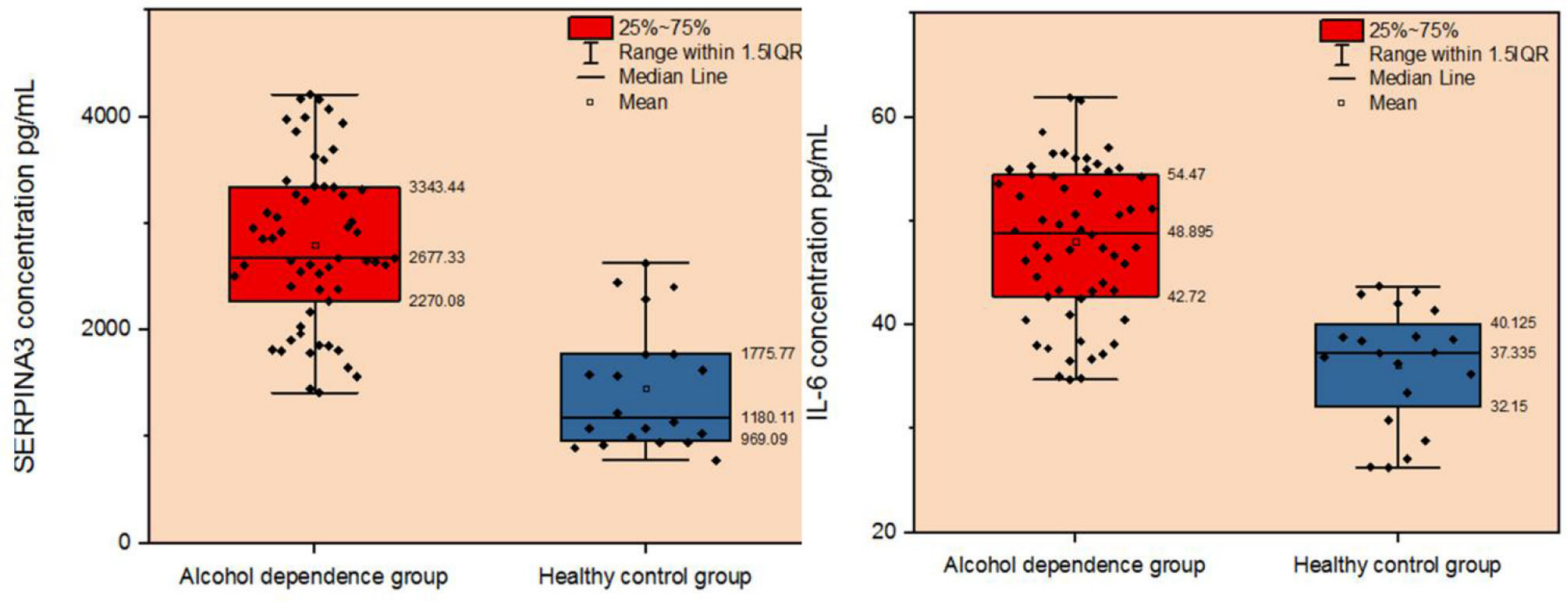
Comparison of plasma SERPINA3 and IL-6 levels between patients with alcohol dependence and the healthy control group.

**Figure 6:**
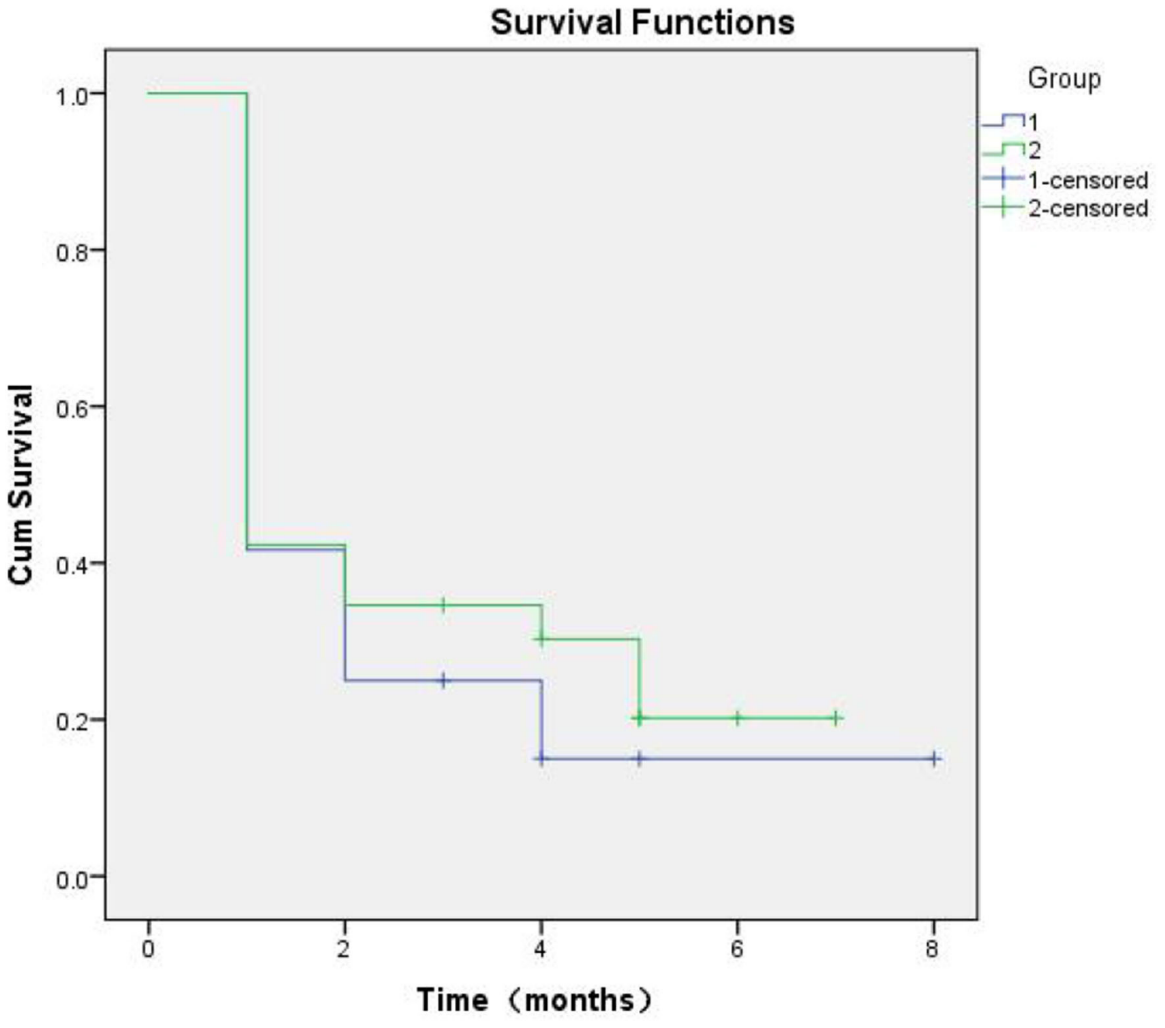
Kaplan-Meier curves based on SERPINA3 levels in patients with alcohol dependence during follow-up of up to 8 months (*P* = 0.489).

In the original article, there was a mistake in Figure 6 as published. We found a problem with the layout of the images. Figure 6 should be Figure 7 in the original article as published.

**Figure 7:**
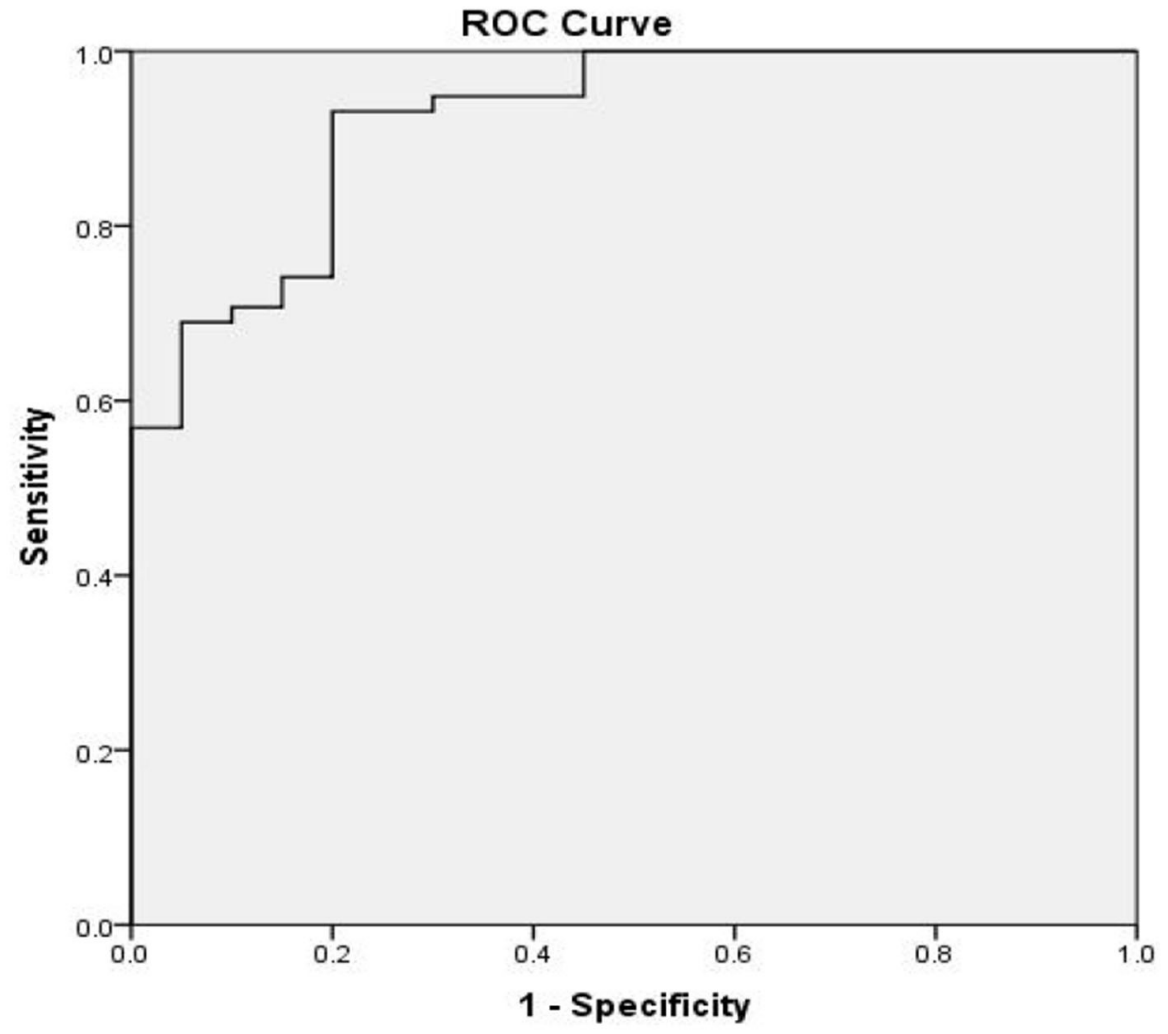
Receiver operator characteristic (ROC) curve based on SERPINA3 levels in patients with alcohol dependence. The area under the curve (AUC) for SERPINA3 was 0.921 (*P* < 0.0001), sensitivity was 93.1%, and specificity was 80.0%.

In the original article, there was a mistake in Figure 7 as published. We found a problem with the layout of the images. Figure 7 should be Figure 8 in the original article as published.

**Figure 8:**
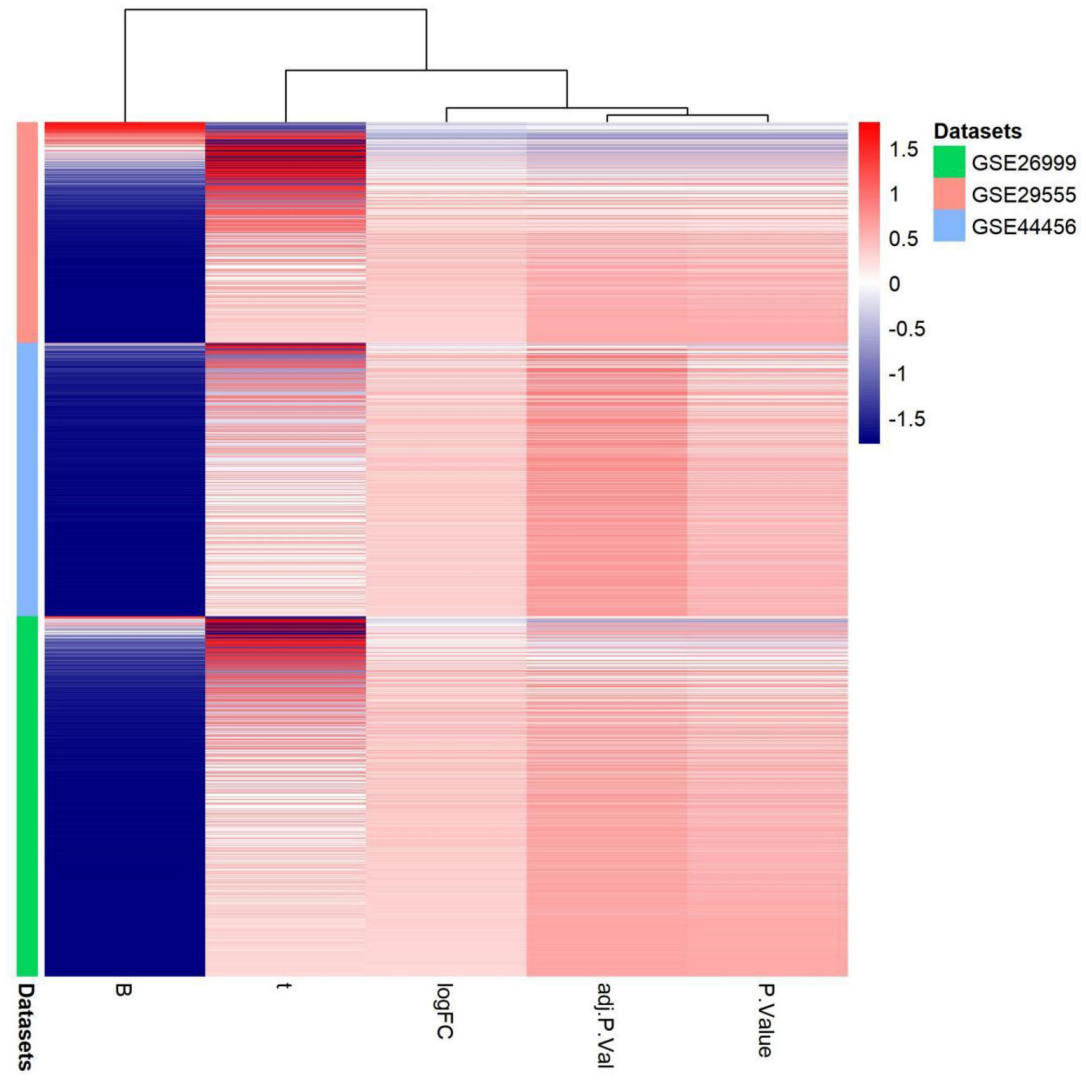
Gene expression patterns in the three gene expression datasets: GSE29555, GSE44456, and GSE62699. Upregulation of genes is marked in red; downregulation of genes is marked in blue.

In the original article, there was a mistake in Figure 8 as published. We found a problem with the layout of the images. Figure 8 should be the chart which was contained inside the Supplemental Material in the original article as published. The corrected figures appear below.

The authors apologize for these errors and state that they do not change the scientific conclusions of the article in any way. The original article has been updated.

## Publisher's Note

All claims expressed in this article are solely those of the authors and do not necessarily represent those of their affiliated organizations, or those of the publisher, the editors and the reviewers. Any product that may be evaluated in this article, or claim that may be made by its manufacturer, is not guaranteed or endorsed by the publisher.

